# Immune-related adverse events and their effects on survival outcomes in patients with non-small cell lung cancer treated with immune checkpoint inhibitors: a systematic review and meta-analysis

**DOI:** 10.3389/fonc.2024.1281645

**Published:** 2024-06-03

**Authors:** Yuxiang Liang, Haidi Xu, Futao Liu, Lei Li, ChenXi Lin, Yaozhong Zhang, Na Wang, Lei Wang

**Affiliations:** ^1^ Department of Thoracic Surgery, The Fourth Hospital of Hebei Medical University, Shijiazhuang, China; ^2^ Department of Infection, The Fourth Hospital of Hebei Medical University, Shijiazhuang, China; ^3^ Cancer Institute, The Fourth Hospital of Hebei Medical University, Shijiazhuang, China

**Keywords:** immune related adverse effects (irAEs), survival & prognosis, non small cell lung cancer (NSCLC), immune check inhibitor (ICI), meta - analysis

## Abstract

**Background:**

The use of immune checkpoint inhibitors (ICIs) has become the standard of care for non-small cell lung cancer. The purpose of this study was to systematically review the literature to determine whether the occurrence of immune-related adverse events (irAEs) following the use of ICIs predicts different clinical outcomes in non-small cell lung cancer (NSCLC).

**Methods:**

Relevant studies from the time of database creation to July 20, 2023, were systematically searched to explore the differences in clinical outcomes in patients with advanced NSCLC with or without irAEs. The outcome indicators included the occurrence of irAEs, progression-free survival (PFS), and overall survival (OS).

**Results:**

25 studies met the inclusion criteria. Of these studies, 22 reported the effect on OS, and 19 reported the effect on PFS. The results showed that for patients with NSCLC, the occurrence of irAEs after receiving immunotherapy showed a statistically significant benefit over the absence of irAEs for OS (HR=0.55,95% CI=0.46–0.65) and PFS (HR=0.55 95% CI=0.48–0.64), but severe irAEs (grades 3–5) were associated with worse OS (HR=1.05, 95% CI=0.87–1.27). Compared with gastrointestinal, lung, and hepatitis, irAEs of the skin and endocrine system tend to predict better OS and PFS.

**Conclusion:**

The occurrence of irAEs, especially mild and early irAEs, indicates better OS and PFS in patients with NSCLC treated with ICIs, irrespective of patient characteristics, type of ICIs, and irAEs. However, Grade 3 or higher toxicities resulted in worse OS.

**Systematic review registration:**

https://www.crd.york.ac.uk/prospero/, identifier CRD42023409444.

## Background

Lung cancer is a common type of thoracic neoplasm that ranks among the forefront of cancers in terms of incidence and mortality. However, its mortality rate has been decreasing annually, owing to early diagnosis and treatment of non-small cell lung cancer (NSCLC) (1).The two primary histological forms of NSCLC are adenocarcinoma (AC) and squamous cell carcinoma (SCC) (2). Locally advanced NSCLC is the initial diagnosis for about 70% of patients with NSCLC, and the 5-year survival rate is less than 3% (3). Previously, patients with advanced NSCLC were usually treated with chemotherapy, radiotherapy, and targeted therapy, but presented poor outcomes with an OS of approximately 12–18 months and a median PFS of only 4–8 months (4, 5).

In contrast, immunotherapy developed by ICIs has revolutionized the treatment strategy for non-small cell lung cancer in recent years (6), mainly including the anti-programmed cell death 1 (PD-1) drugs, Nivolumab and Pembrolizumab, and the anti-programmed cell death ligand 1 (PD-L1) drugs, Atezolizumab and Durvalumab (7). It can be leveraged to leverage the intrinsic immune response against tumor antigens by taking away the inhibitory effect that antigen-presenting cells (APCs) have on T-cell activation. Nevertheless, these drugs have the potential to stimulate T-cell attack on self-antigens through the same mechanism, leading to a clinical manifestation of distinct toxicities known as irAEs (5).With the widespread use of ICIs, irAEs such as skin damage, myocarditis, hepatitis, colitis, endocrine disorders, inflammatory arthritis, and pneumonitis, have been widely reported (8, 9). Most irAEs tend to be mild and self-limiting, while 2–18% of patients present with grade 3 or 4 irAEs that require prompt recognition and management (10).

The correlation between irAEs and improved clinical outcomes was first observed in patients with melanoma and, in recent years, with the widespread use of ICIs in NSCLC (11), several studies have shown that the occurrence of irAEs after the use of ICIs correlates with clinical outcome indicators. A systematic review of 30 studies revealed that irAEs, such as pulmonary, thyroid and gastrointestinal diseases, were associated with improved OS and PFS in patients with NSCLC and melanoma (12). However, the review did not provide separate data analysis for NSCLC patients. A robust and precise systemic review is required to evaluate the association between irAEs occurrence and the efficacy of ICIs in advanced NSCLC patients. Herein, we conducted a systematic review and meta-analysis to investigate whether OS and PFS are associated with the occurrence of irAEs in patients with advanced non-small cell lung cancer using ICIs.

## Methods

### Study objectives and inclusion criteria

The purpose of this systematic review was to summarize and provide a qualitative and quantitative review in the form of a meta-analysis to address the following research question: “Is there an improvement in survival among patients diagnosed with non-small cell lung cancer and treated with ICIs who develop irAEs?” We used the population-intervention-comparison-outcomes-study design (PICOS) framework to construct the research question and its corresponding literature search. This systematic review and meta-analysis was registered in the International Prospective Register of Systematic Reviews (CRD42023409444).

### Literature search strategy

The study was based on the Preferred Reporting Items for systematic reviews and meta-analyses (PRISMA) statement for literature search, study inclusion, extraction of data, and consolidation of results. We identified eligible studies from databases, such as PubMed, ISI Web of Science database, and Cochrane Library from the time of its creation to July 20, 2023 (**Supplementary Table S1**). The search terms included the following subject terms and free terms: ((“immune checkpoint inhibitor” OR “Checkpoint Inhibitors, Immune” OR “immune checkpoint blockade” OR “Checkpoint Inhibitor, Immune” OR “immune checkpoint blockades” OR “Checkpoint Blockers, Immune” OR “Checkpoint Blockade, Immune” OR “Immune Checkpoint Inhibition” OR “Checkpoint Inhibition, Immune” OR “PD-L1 Inhibitors” OR “PD L1 Inhibitors” OR “PD-L1 Inhibitor” OR “PD L1 Inhibitor” OR “Programmed Death-Ligand 1 Inhibitors” OR “Programmed Death Ligand 1 Inhibitors” OR “PD-1-PD-L1 Blockade” OR “Blockade, PD-1-PD-L1” OR “PD 1 PD L1 Blockade” OR “PD-1 Inhibitors” OR “PD 1 Inhibitors” OR “PD-1 Inhibitor” OR “Inhibitor, PD-1” OR “PD 1 Inhibitor” OR “Programmed Cell Death Protein 1 Inhibitor” OR “Programmed Cell Death Protein 1 Inhibitors”) OR (“nivolumab” OR “pembrolizumab” OR “atezolizumab” OR “durvalumab” OR “avelumab” OR “ipilimumab” OR “cemiplimab” OR “Tislelizumab” OR “camrelizumab” OR “toripalimab”)) AND (“Carcinoma, Non-Small-Cell Lung” OR “Lung Carcinoma, Non-Small-Cell” OR “Non-Small-Cell Lung Carcinomas” OR “Non-Small Cell Lung Cancer”) AND ((“immune-related”) AND (“adverse” OR “adversely” OR “adverses”) AND (“event” OR “event s” OR “events”)).

### Selection and data extraction

Two authors (HD and LL) independently retrieved the available literature to identify eligible studies. The studies were chosen based on the following criteria: (a) studies that only included patients with non-small cell lung cancer; (b) the primary efficacy outcomes with the occurrence of irAEs, progression-free survival (PFS), and overall survival (OS). (c) randomized controlled trials (RCTs) or retrospective experiments comparing non-small cell lung cancer patients with and without immune-related adverse events after immunotherapy. The exclusion criteria were as follows: (a) studies reporting incomplete or inconsistent outcomes; and (b) duplicate studies, studies reporting animal experiments, case reports, cohort studies, and review articles. Once the final set of included studies was identified, data were extracted independently by three authors (HD, LL, and CX) using a pre-designed form implemented in Microsoft Excel 2010 version, and any disagreements were resolved through consensus discussions.

The following information was collected from each study: first author, year of publication, study type, study population characteristics, immune checkpoint inhibitor type, total percentage of patients with irAEs, percentage with grade 1–2 irAEs, percentage with grade 3–5 irAEs, landmark analysis and the HR associated with prognostic outcomes (OS and/or PFS). If the HR and 95% CI were not directly provided in the original article, summary time-to-event data were included in the meta-analysis (13). In addition, if available, a multivariate analysis was preferable because it considers possible confounding factors (14).

### Data analysis

All analyses were conducted using the STATA statistics software V16.0 and Review manager V5.3. First, the clinicopathological and prognostic significance of the occurrence of irAEs in locally advanced NSCLC was summarized using the HR and its associated 95% confidence interval (CI) as impact indicators. When available, the multivariate adjusted risk was used in each study. All eligible studies were included in the analyses. In addition, we tested for publication bias using funnel plots of Egger’s and Begg’s tests. If the *P*-value of the test was less than 0.05, it indicated publication bias. In addition, Egger’s test is usually considered more sensitive than Begg’s test (15). We chose the results of the Egger’s test if they were inconsistent.

The Newcastle-Ottawa scale (NOS; range, 0–9)1 was used to assess the quality of each study. A score of > 6 was considered as high quality. Studies with a score ≤6 were excluded.

We evaluated the statistical heterogeneity among the studies using the X^2^-based Q test and I^2^ statistics. When *P* > 0.05 for the Q test and I^2^<50%, the fixed-effect model with the Mantel-Haenszel technique was applied; otherwise, the random-effect model with the inverse-variance method was utilized, and the pooled HRs and 95% CIs for all included studies were calculated. Subgroup and meta-regression analyses were used to explore heterogeneity, if necessary. Sensitivity analysis was also conducted to ensure the stability of the results; all statistical analyses were two-sided, and a *P* value less than 0.05 was considered statistically significant.

## Results

### Study selection

**Figure 1:**
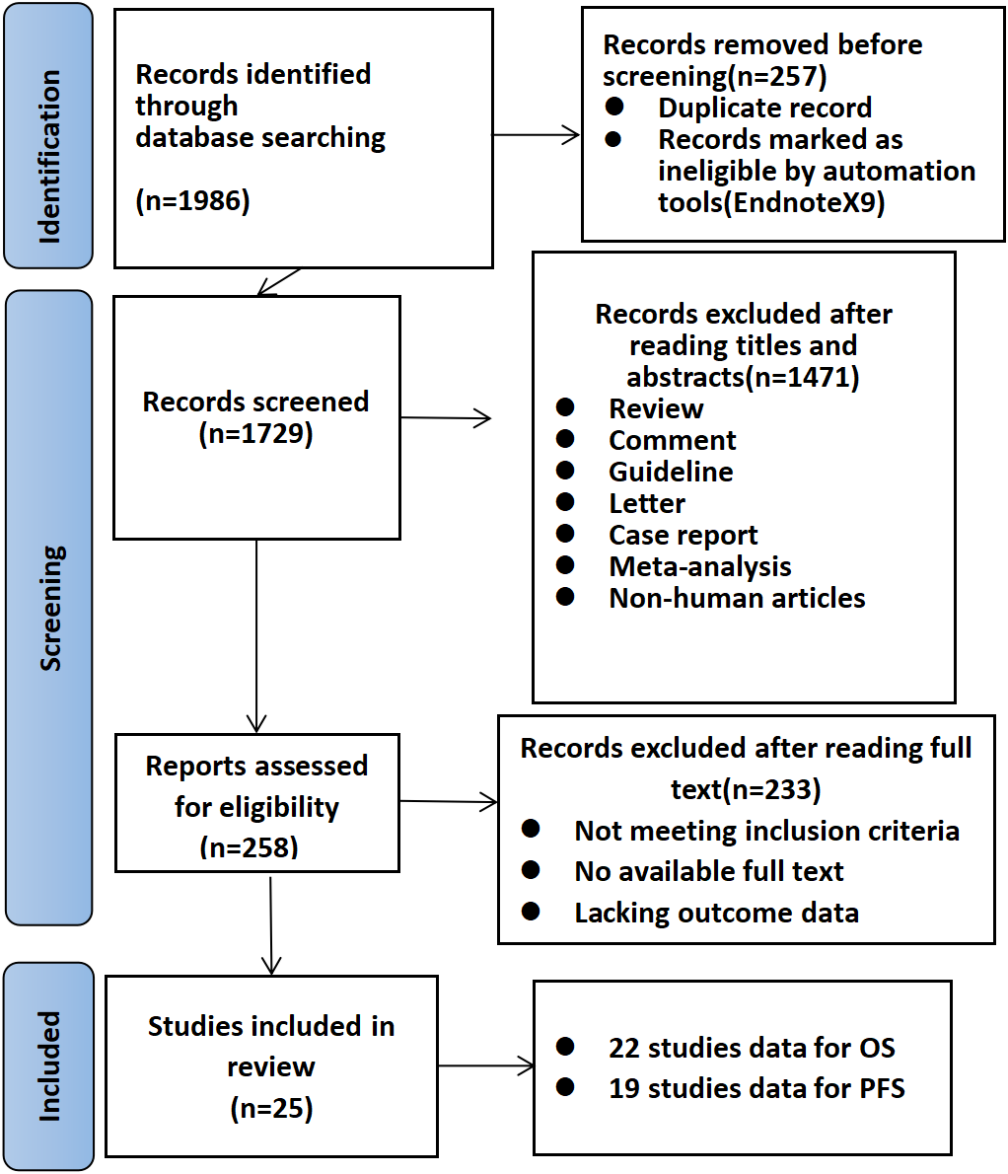
Flow diagram of the literature search and article evaluation process.

**Figure 2:**
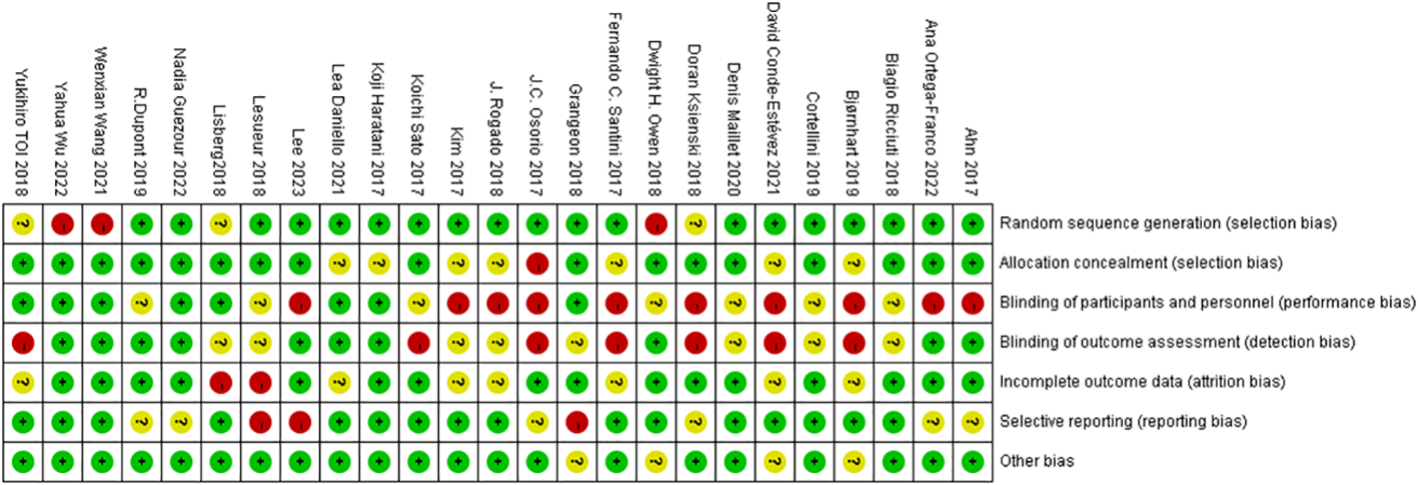
Bias from seven key sources assessed with the Cochrane Risk Bias Assessment Tool, with green representing low risk, yellow representing medium risk, and red representing high risk.

The online database search identified 1986 studies. The removal of duplicate and irrelevant articles left 1729 records. Removing nonhuman and nonclinical trial articles resulted in 258 abstracts that met the screening criteria. The full texts of these 258 articles, including additional appendices, were reviewed. Of the 258 studies, 25 met all the inclusion criteria (16–40) detailed data are provided in **Table 1**. 22 of these studies reported effects on OS (16–21, 23–28, 30–37, 39, 40), and 19 reported effects on PFS (16–18, 21–24, 26–29, 31, 32, 34–37, 39, 40). We have also summarized the incidence and effectiveness of different types of irAEs in a new table (**Supplementary Table S2**). A PRISMA flowchart was developed to summarize the study selection process along with a quality evaluation of the included literature (**Figures 1**, **2**). The incidence of adverse reactions after receiving immunotherapy was extracted from each study, including the overall incidence and the incidence of grade 1–2 mild and grade 3–5 severe. The hazard ratios and 95% confidence intervals for OS and PFS for the occurrence of irAEs compared to the absence of irAEs were extracted, and 7 studies by Denis et al. (18) and Nadia et al. (25) provided data on the association between OS and severe immune adverse reactions.

**Table 1:** Summary of study characteristics and efficacy of all included cohort studies.

Author	Study type	immune checkpoint inhibitor type	Initial irAEs			Overall survival hazard ratio (95% CI)	Progression-free survival hazard ratio (95% CI)	Grade 3–5 irAEs Overall survival hazard ratio (95% CI)	Landmark analysis	Trial design
			Total	Low grade (1–2)	High grade (3–5)					
Koji Haratani 2018 (23)	M	PD-1(Nivolumab)	69/134	57/134	12/134	0.28 (0.10–0.79)	0.52 (0.29–0.96)	NA	6 weeks	R
J.C. Osorio 2017 (21)	M	PD-1(Pembrolizumab)	10/48	9/48	1/48	0.29 (0.09–0.93)	0.58 (0.27–1.25)	NA	NA	P
Doran Ksienski 2019 (33)	S	PD-1(Pembrolizumab, Nivolumab)	100/230	77/230	23/230	0.85 (0.50–1.42)	NA	2.29 (1.05–4.99)	6 weeks	R
R.Dupont 2020 (26)	M	PD-1(Nivolumab)	58/191	49/191	9/191	0.58 (0.41–0.82)	0.36 (0.26–0.50)	NA	12 weeks	R
Yukihiro TOI 2018 (29)	S	PD-1(Nivolumab)	42/70	41/70	1/70	NA	0.43 (0.21–0.88)	NA	NA	R
Wenxian Wang 2022 (27)	S	PD-(L)1	79/222	59/222	20/222	0.76 (0.53–1.09)	0.65 (0.48–0.88)	NA	6 weeks	R
Koichi Sato 2018 (22)	S	PD-1(Nivolumab)	11/38	10/38	1/38	NA	0.10 (0.02–0.50)	NA	60 days	R
Lea Daniello 2021 (24)	M	PD-(L)1	232/894	121/894	111/894	0.38 (0.27–0.53)	0.65 (0.48–0.88)	NA	14 weeks	R
Biagio Ricciuti 2019 (16)	S	PD-1(Nivolumab)	85/195	70/195	15/195	0.55(0.33–0.92) 0.4(0.26–0.59)	0.69(0.45–1.05) 0.48(0.34–0.69)	NA	6 weeks 12 weeks	R
Ana Ortega-Franco 2022 (30)	S	PD-1	47/113	33/113	14/113	0.51 (0.31–0.84)	NA	NA	NA	R
David Conde-Estévez 2021 (17)	S	PD-(L)1	31/70	26/70	5/70	0.46 (0.25–0.85)	0.63 (0.33–1.20)	NA	NA	R
Denis Maillet 2020 (18)	M	PD-(L)1	104/304	80/304	24/304	0.50 (0.36–0.69)	0.58 (0.43–0.78)	1.10 (0.57–2.12)	NA	R
Yahua Wu 2022 (28)	S	PD-(L)1	45/101	37/101	8/101	0.52 (0.29–0.93)	0.57 (0.34–0.96)	NA	12 weeks	R
Nadia Guezour2022 (25)	M	PD-(L)1	119/201	83/201	36/201	0.48 (0.19–1.23)	NA	3.00 (1.80–5.00)	NA	P
Fernando C. Santini 2017	M	PD-(L)1	68/482	35/482	33/482	0.24 (0.09–0.62)	0.46 (0.21–1.01)	NA	12 weeks	R
J. Rogado 2019 (20)	S	PD-1(Pembrolizumab, Nivolumab)	40/77	30/77	10/77	1.10 (0.70–1.73)	NA	2.30 (1.40–3.78)	NA	R
Kim 2017 (32)	S	PD-1(Pembrolizumab, Nivolumab)	19/58	19/58	0/58	0.11 (0.01–0.92)	0.38 (0.17–0.85)	NA	NA	R
Ahn 2019 (37)	S	PD-1(Pembrolizumab, Nivolumab)	73/155	65/155	8/155	0.40(0.25–0.65)	0.36(0.23–0.56)	NA	6 weeks	R
Bjørnhart 2019 (38)	S	PD-(L)1	NA	NA	25/118	NA	NA	0.47 (0.21–1.05)	NA	R
Cortellini 2019 (39)	M	PD-1(Pembrolizumab, Nivolumab)	231/559	181/559	50/559	0.55 (0.41–0.72)	0.59 (0.47–0.76)	0.53 (0.41–0.69)	6 weeks	R
Grangeon 2019 (40)	S	PD-(L)1	124/270	NA	NA	0.29 (0.18–0.46)	0.42 (0.32–0.57)	NA	NA	R
Lee 2023 (34)	M	PD-L1(Atezolizumab)	275/300	139/300	136/300	0.78 (0.63 - 0.97)	0.87 (0.70 -1.07)	NA	NA	P
Lesueur 2018 (35)	M	PD-1(Nivolumab)	62/104	52/104	10/104	0.64 (0.38–1.09)	0.66 (0.43–1.1)	NA	NA	R
Lisberg 2018 (36)	S	PD-1(Pembrolizumab)	28/97	NA	NA	0.72 (0.49–1.05)	0.75 (0.56–0.99)	NA	NA	R
Owen 2018 (19)	S	PD-(L)1	27/91	21/91	6/91	0.90(0.72–1.13)	NA	1.20 (0.76–1.92)	3 months	R

ICIs, immune checkpoint inhibitors; irAEs, immune-related adverse events; M, Multicenter study; S, Single-center study; PD-1, programmed cell death protein-1; PD-L1, programmed cell death protein ligand-1; P, Prospective; R, Retrospective; NA, not applicable.

### Correlation between irAEs and OS

**Figure 3:**
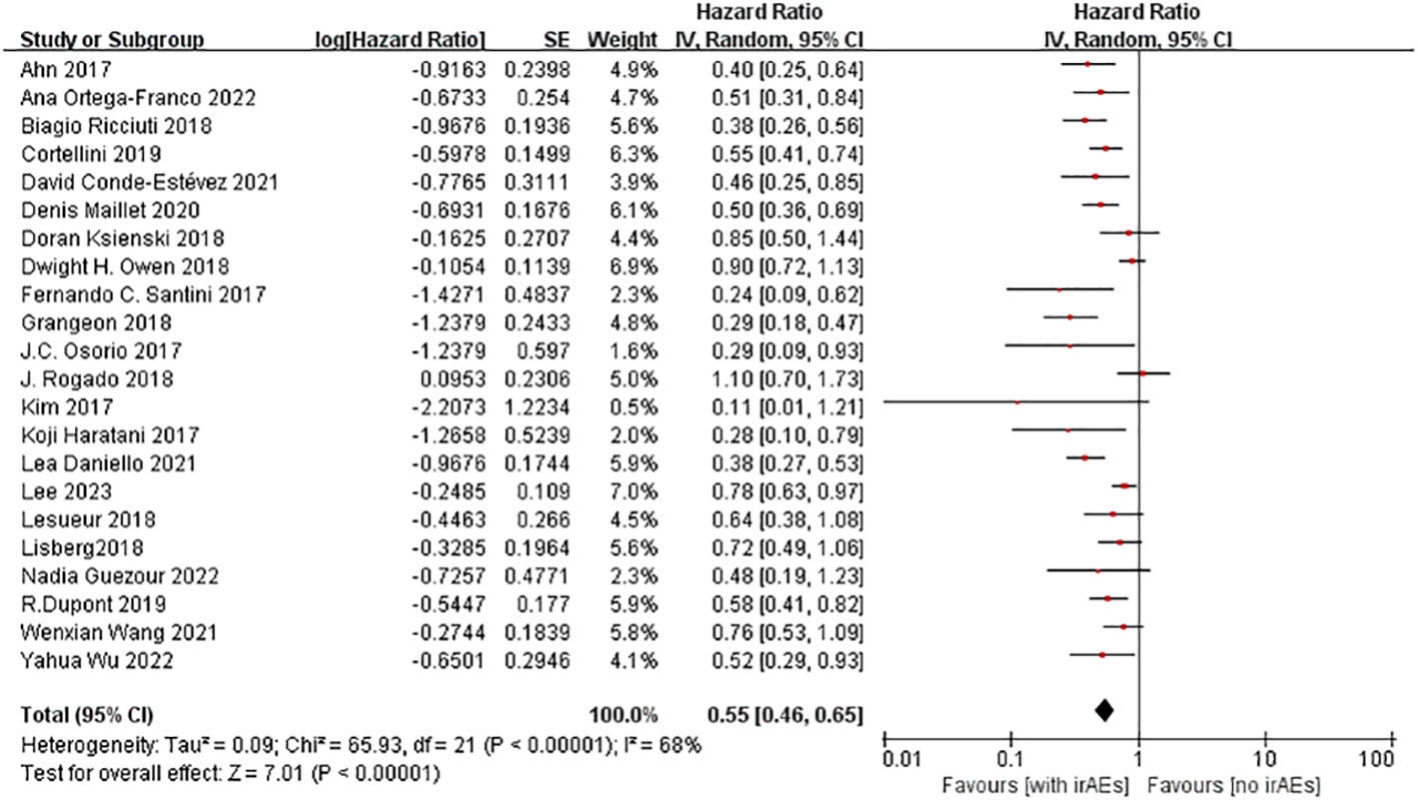
Forest plot of OS in NSCLC patients.

**Figure 4:**
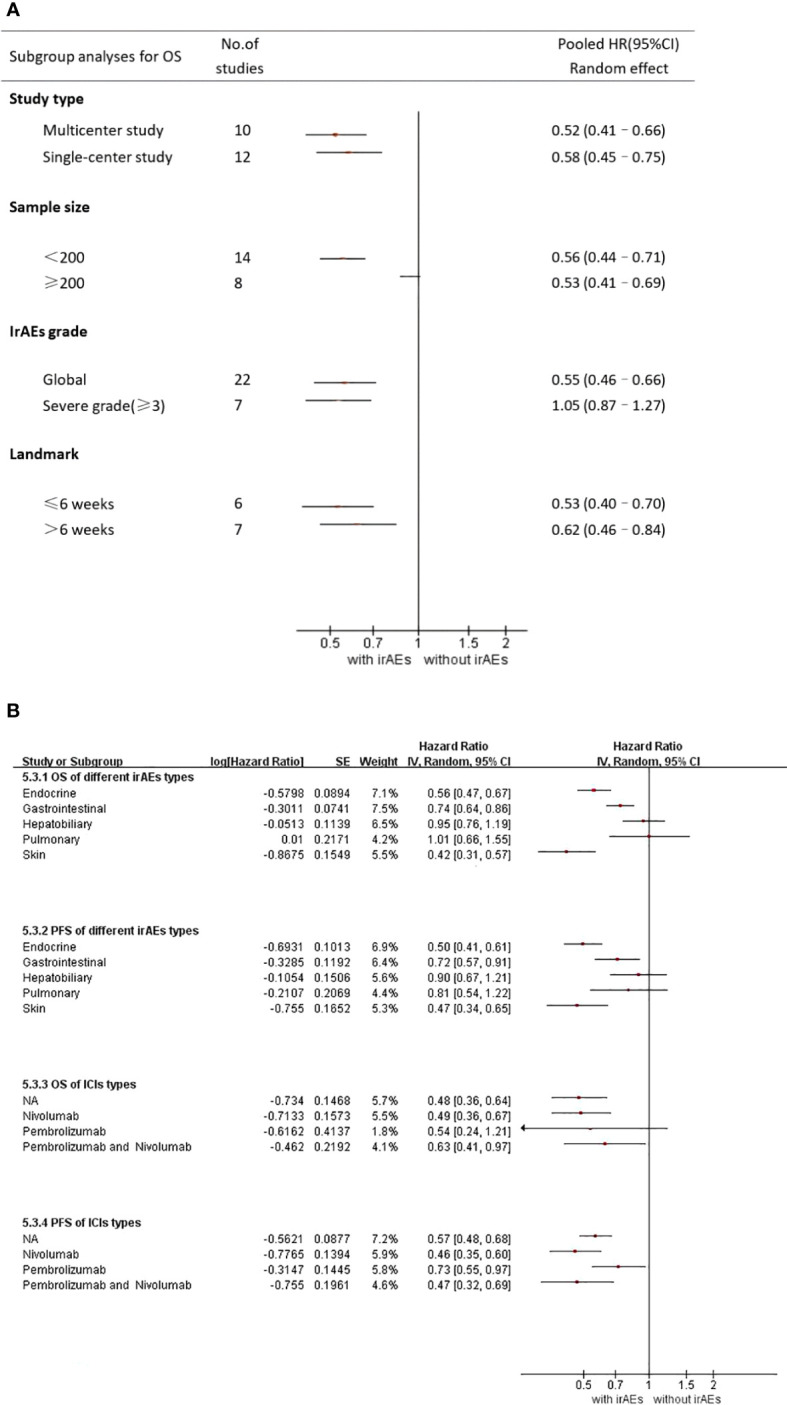
**(A)** Subgroup analysis of the association between irAEs and OS **(B)** Subgroup analysis of irAEs types and ICI types NA, not applicable.

A total of 22 studies with OS data were obtained (16–21, 23–28, 30–37, 39, 40), and overall hazard ratios, including the occurrence and absence of irAEs, were observed. The occurrence of irAEs in patients with advanced NSCLC treated with immunotherapy reduced the risk of death by 45% when compared to the non-occurrence of irAEs(HR=0.55, 95% CI=0.46–0.65; **Figure 3**). The percentage of the total heterogeneity/total variability was high (I^2^ = 68%). In addition, from the subgroup analysis, we found that some of the subgroups did not have significant differences, such as group of sample size and ICI types but other subgroups showed subtle differences. For example, in **Figure 4A**, we showed that multicenter studies (HR=0.52, 95% CI=0.41–0.66)predict better OS than single-center studies (HR=0.58, 95% CI=0.45–0.75). Also in the landmark analysis, we concluded that the clinical outcome of patients with irAES at less than or equal to 6 weeks(HR=0.53, 95% CI=0.40–0.70) was better than that at more than 6 weeks(HR=0.62, 95% CI=0.46–0.84).

7 studies by Denis et al. reported a relationship between severe adverse immune reactions (grade 3 irAEs) and OS (18–20, 25, 33, 38, 39). In **Figure 4A**, we observed the association between grade 3–5 irAEs and OS (HR = 1.05, 95% CI = 0.87–1.27). This illustrates that severe adverse immunotherapeutic effects (grade 3 irAEs) lead to decreased OS in patients with advanced NSCLC and are detrimental to patient prognosis.

As shown in **Supplementary Table S2**, irAEs mainly occurred in the skin, digestive system, pulmonary, endocrine system, and hepatobiliary system. Skin and endocrine irAEs were the most common. In addition, 11 studies reported the relationship between different types of irAEs and survival (16, 17, 21–24, 26, 32, 37, 39, 40). From **Figure 4B**, we concluded that skin irAEs (HR=0.42 95% CI=0.31–0.57)and endocrine irAEs(HR=0.56 95% CI=0.47–0.67) indicate better prognosis than other irAEs. However, pulmonary irAEs (HR=1.01 95% CI=0.66– 1.55) was a relatively poor type.

### Correlation between irAEs and PFS

**Figure 5:**
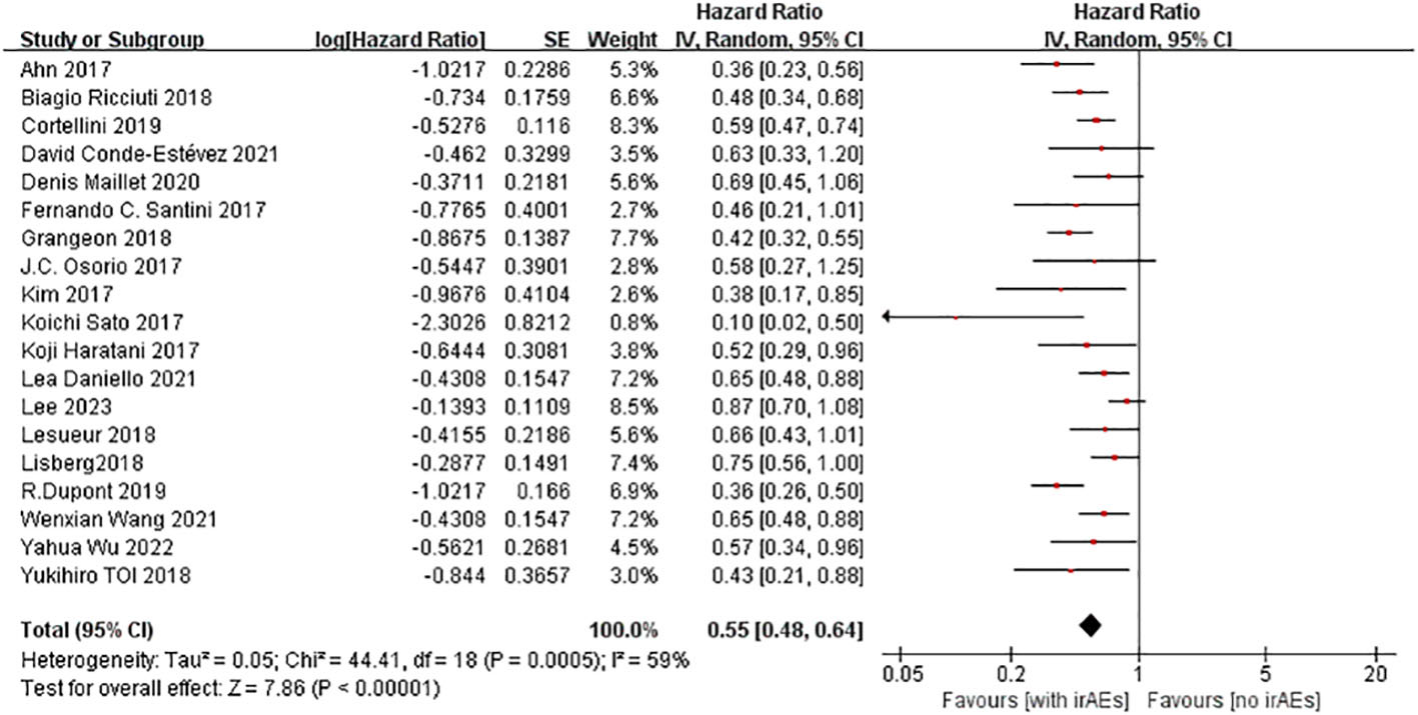
Forest plot of PFS in NSCLC patients.

19 studies reported PFS (16–18, 21–24, 26–29, 31, 32, 34–37, 39, 40) with a Random-effects model for the 2-group comparison using HR as an effect indicator. The results of the meta-analysis (**Figure 5**) showed that the risk of disease progression with irAEs in patients with advanced NSCLC receiving ICIs was 45% of that in patients without irAEs. This difference was statistically significant (HR=0.55; 95% CI = 0.48–0.64). This result suggested that the occurrence of irAEs in patients with advanced NSCLC receiving ICIs prolongs the PFS of their disease.

For different types of irAEs, skin irAEs (HR=0.47 95% CI=0.34–0.65) and endocrine irAEs (PFS : HR=0.50 95%CI=0.41–0.61) indicated better PFS than other irAEs. However, hepatobiliary irAEs (HR=0.78 95% CI=0.53–1.14) and pulmonary irAEs(PFS : HR=0.81 95% CI=0.54–1.22)were not significantly associated with a favorable PFS.

Different from the results of OS, in the subgroup analysis of ICI types, we showed obvious differences in the study results of Pembrolizumab(HR=0.73 95% CI=0.55–0.97) and Nivolumab(HR=0.46 95% CI=0.35–0.60), but we believed that it may be related to the heterogeneity caused by the small sample size of Pembrolizumab. Further clinical studies are needed to prove this.

### Sensitivity analysis and publication bias

**Figure 6:**
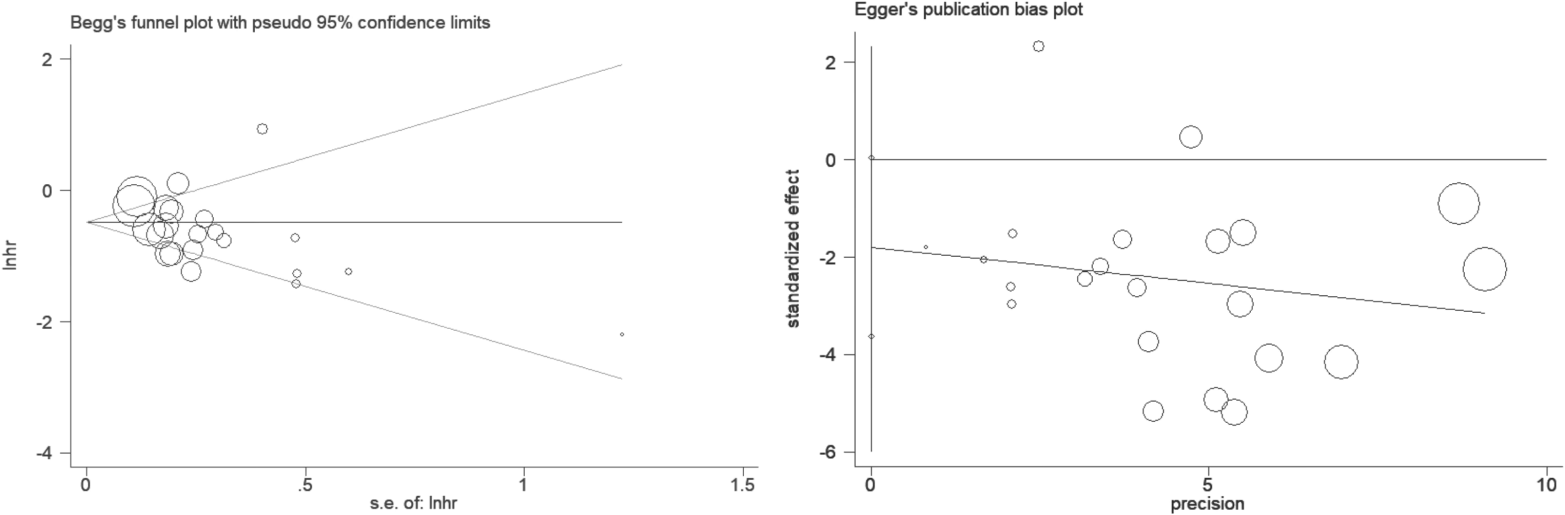
Egger ‘ s test and Begg ‘ s test for OS.

**Figure 7:**
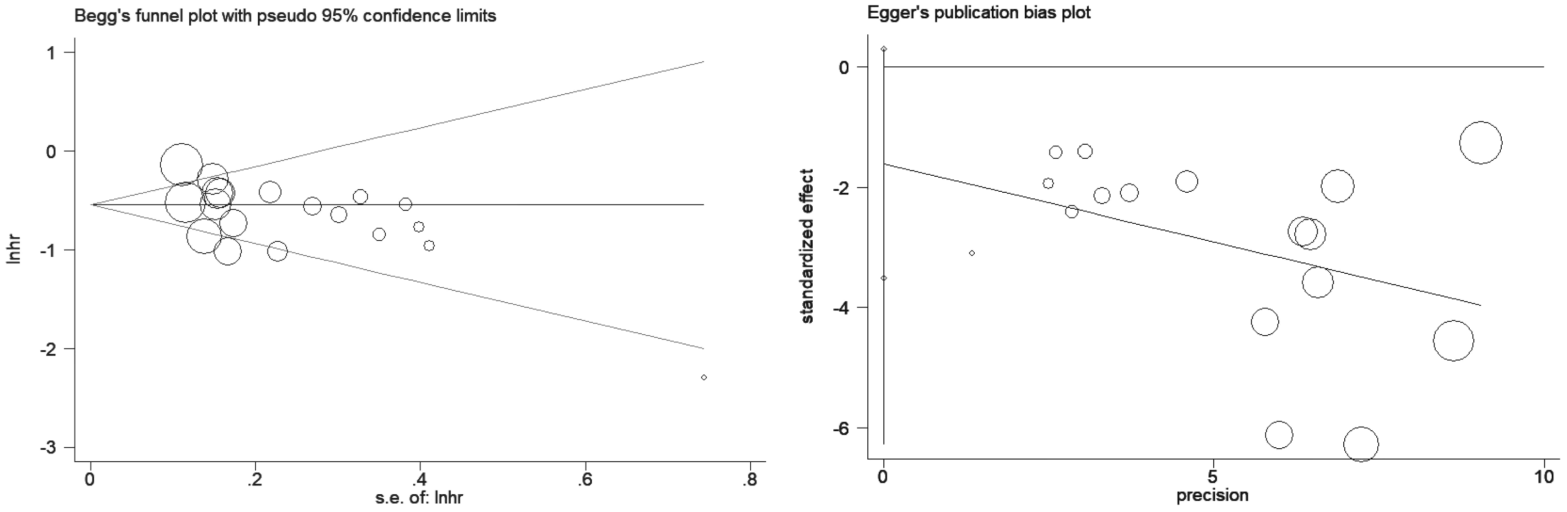
Egger ‘ s test and Begg ‘ s test for PFS.

In the sensitivity analysis, regardless of whatever trial was removed, the combined results for OS and PFS remained significant, showing that there was a strong correlation between the incidence of irAE and the effectiveness of ICIs in NSCLC patients. Publication bias in this meta-analysis was indicated by Egger’s and Begg’s tests (**Figures 6**, **7**; **Table 2**). The results revealed no significant publication bias in the included studies.

**Table 2:** Result of publication bias.

Item	Egger’s test	Begg’s test
OS	0.195	0.063
PFS	0.074	0.093

## Discussion

Given the emergence of immune checkpoint inhibitors (ICIs) as a therapeutic strategy for non-small cell lung cancer (NSCLC) in recent years, there has been a concerted effort to identify reliable biomarkers that can predict response to ICIs through intensive research (40). Early clinical studies exploring immunotherapy have suggested a potential association between the occurrence of immune-related adverse events (irAEs) and prognosis in NSCLC patients. A systematic review performed by Zhou et al. summarized the studies investigating the association between irAEs and ICIs efficacy in patients with cancer (12). It was reported that irAEs predicted better OS and PFS regardless of tumor type. However, whether for global OS or PFS, as well as different types of irAEs, the relevant data of NSCLC were not listed separately in the article. Therefore, we conducted this meta-analysis to investigate whether the presence of irAEs impacts overall survival (OS) or progression-free survival (PFS) in advanced NSCLC patients. Our study corroborated previous findings with 25 literature sources and an enrollment of 5213 patients. The overall incidence rate of irAEs was found to be 35.6%, with mild immune adverse reactions (Grade 1–2) occurring at a rate of 25.1% and severe immune responses at a rate of 9.7%. The hazard ratio for total OS was calculated as 0.55, with a confidence interval (CI) of 0.46–0.65, while the hazard ratio for total PFS was determined as 0.55 with a CI of 0.48–0.64; these results unequivocally demonstrated that the occurrence of irAEs, particularly mild and early ones, conferred benefits on both OS and PFS outcomes in advanced NSCLC patients.

However, our understanding of the mechanisms behind the genesis of irAEs is still lacking. Several mechanisms have been suggested to contribute to the occurrence of irAEs, according to prior reports. These include homologous antigens/epitopes present in both normal tissues and tumor cells, autoantibody production, direct binding of ICIs to immune checkpoint molecules expressed on the surface of normal cells or complement activation, and elevated levels of inflammatory factors (41). Therefore, the most likely mechanism for the development of irAEs could be the abnormal activation of T cells that are specific to a target tissue, and that activation of T cells would cause the production of inflammatory components. For example, PD-(L)1 inhibitors act in the T-cell effector phase, mainly activating T cells in peripheral tissues, thereby increasing the specificity of irAEs (8).

In general, irAEs are mild and manageable (42). As reported previously, most irAEs are cutaneous disorders, with rashes being the most prevalent (17). For example, reactive cutaneous capillary endothelial proliferation (RCCEP) is the most common skin-related immune-related adverse reaction to the PD-1 inhibitor camrelizumab, with an incidence of approximately 78.8% (834/1059) and occurs mainly in the superficial skin of the face and trunk, and is characterized by capillary hyperplasia in the skin dermis. RCCEP mostly appears 2–4 weeks after the first dose of ICIs, does not increase in size at 3–4 months, and can atrophy, recede, or become necrotic 1–2 months after the termination of ICIs. Very few patients present in the oral, nasal, or oculofacial mucosa; however, to date, it has not occurred in the respiratory and gastrointestinal mucosa. Therefore, RCCEP can be used as a clinical indicator to predict the efficacy of camrelizumab monotherapy (43). In addition to skin diseases, irAEs usually manifest as thyroid disease, colitis, pneumonitis, and hepatitis (17). Zhou et al. reported that the occurrence of endocrine and skin irAEs predicted better OS and PFS. Nevertheless, the occurrence of pulmonary and hepatobiliary irAEs was not significantly associated with favorable OS and PFS (12). This is consistent with our findings. We supposed that the observed outcome may be attributed to other irAEs such as thyroid and skin diseases, which typically have a self-limiting nature and milder symptoms. However, checkpoint inhibitor pneumonitis(CIP) could lead to various degrees of lung damage, ranging from the acute stage (acute interstitial pneumonia [AIP]) to the tissue stage (histological pneumonia [OP]) and the fibrotic stage (nonspecific interstitial pneumonia [NSIP]). The majority of CIP cases represent severe irAEs necessitating high doses of oral or parenteral steroids. A comprehensive retrospective cohort study revealed that 86% of patients with CIP demonstrated improvement following corticosteroid therapy. However, a notable 14% of CIP patients did not show any signs of improvement post-treatment and exhibited limited response to alternative immunosuppressants, ultimately leading to unfavorable patient outcomes (44, 45).

For the correlation between severe immune adverse reactions (grade 3–5) and clinical outcomes, we observed that grade 3–5 irAEs were unfavorable for OS (HR=1.05, 95% CI=0.87–1.27), while the sample size for PFS results was too small; only Denis et al. showed that grade 3–5 irAEs were also favorable for PFS (HR=0.66, 95% CI=0.40–1.08) (18). This might be related to the following reasons. Large doses of steroids are needed for early severe irAEs, which reduces the effectiveness of ICIs. Furthermore, immunotherapy may be interrupted by severe irAEs, which could impact the prognosis. In addition, a few studies have suggested that the interaction between tumor cells and T cells, cytokines, and antibodies may be linked to significant adverse events and worse clinical outcomes. When using PD-1/PD-L1 inhibitors, macrophage regulatory T cells can exert antibody-dependent cellular phagocytosis through Fc receptors (FcR) and T cell antigen receptors, stimulating the growth of certain cells while suppressing the proliferation of other cells. The anti-tumor activity of immune cells exerts a potent tumor-promoting effect (27, 46).

Numerous studies have shown that systemic immunotherapy should be discontinued when grade 3–5 irAEs occurs (25, 47) because severe adverse effects such as pneumonitis and thrombocytopenia may directly lead to patient death, which may affect prognostic outcome indicators (27). In addition, there is a meta-analysis on Tocilizumab that allows continuation of immunotherapy in the presence of severe irAEs with significant efficacy, but this still needs to be confirmed in controlled prospective studies (48). Petrelli (49) confirmed a significantly worse prognosis in patients receiving steroids during treatment with ICIs (HR = 1.54, 95% CI:1.24–1.91, p < 0.0001). In addition, Wang (27) showed that regardless of the early or late appearance of irAEs, patients who did not require systemic glucocorticoid therapy affecting thyroid function, skin, and other adverse effects had a better prognosis than patients with pneumonitis abnormal liver function, and other adverse effects requiring systemic glucocorticoid therapy because they required less frequent and cumulative measures of steroids. However, R. Dupont reported that anti-PD1 outcomes are similar in patients treated with steroids for irAEs and patients experiencing irAEs who do not require the use of steroids. Additionally, they discovered that PFS was negatively impacted by steroids used to manage irAEs, but not OS (48). We hypothesize that this might be connected to the kind of steroid medication, when treatment is administered, and the kind of tumor, but more investigation is required to validate this. In addition, it has been proposed that the presence of 2 irAEs may suggest better clinical outcomes than the occurrence of 1 irAE (19).

## Limitations

This meta-analysis has several limitations, and it is preferable to rely on published outcomes rather than individual patient data. Using a random-effects model, we assumed that these 25 studies represented a random sample of all hypothetical studies wherein there was a treatment effect on the outcome measures. Thus, the pooled effect represents the average effect in the entire study population. Second, chemoimmunotherapy combination therapy has become a routine treatment for NSCLC, and the effects of the type, dose, and frequency of chemotherapeutic agents on clinical outcome indicators are also under consideration. Moreover, although quality assessment was performed, most of the studies were retrospective, and the included studies for PD-1 drugs were mainly focused on Pembrolizumab and Nivolumab; there were fewer data for other PD-1 drugs, such as Camrelizumab or Tislelizumab. Therefore, it is difficult to apply the study findings to all patients.

## Conclusion

Overall, the occurrence of irAEs, particularly mild and early irAEs, positively correlated with PFS and OS in patients with advanced NSCLC treated with ICIs. However, irAEs of grade 3 and above resulted in a poorer OS. For different irAEs types, skin and endocrine irAES predicted better OS and PFS than pulmonary and hepatobiliary irAEs. As the use of ICIs continues to expand, early detection and management of these irAEs will become even more important to maximize the duration of treatment while minimizing toxicity to patients. Simultaneously, we think it’s critical to find indicators that can recognize and forecast adverse reactions, identify people at risk for severe adverse reactions, and evaluate the prognosis of patients in advance.

## Data availability statement

The original contributions presented in the study are included in the article/**Supplementary Material**. Further inquiries can be directed to the corresponding authors.

## Author contributions

YL: Conceptualization, Data curation, Formal analysis, Investigation, Methodology, Software, Writing – original draft, Writing – review & editing. HX: Data curation, Methodology, Writing – review & editing. FL: Methodology, Supervision, Writing – review & editing. LL: Methodology, Software, Writing – review & editing. CL: Data curation, Methodology, Writing – review & editing. YZ: Methodology, Supervision, Writing – review & editing. NW: Formal analysis, Resources, Writing – review & editing. LW: Resources, Writing – review & editing.
