# Supplementary material for: Immune-related adverse events and their effects on survival outcomes in patients with non-small cell lung cancer treated with immune checkpoint inhibitors: a systematic review and meta-analysis
Source: Front Oncol. 2024 Jun 3;14:1281645. doi: 10.3389/fonc.2024.1281645 (PMC11180722; doi:10.3389/fonc.2024.1281645)
Supplement: Supplementary file 1 [file Table_1.docx]

**Table S1.**

**Pubmed Search strategy.**

| **# No** | **Searches** |
| --- | --- |
| Part I: Immune checkpoint inhibitor | |
| **1** | “immune checkpoint inhibitor”[Mesh] OR “Checkpoint Inhibitors, Immune”[Title/Abstract] OR “immune checkpoint blockade”[Title/Abstract] OR “Checkpoint Inhibitor, Immune”[Title/Abstract] OR “immune checkpoint blockades”[Title/Abstract] OR “Checkpoint Blockers, Immune”[Title/Abstract] OR “Checkpoint Blockade, Immune[Title/Abstract]” OR “Immune Checkpoint Inhibition[Title/Abstract]” OR “Checkpoint Inhibition, Immune[Title/Abstract]” OR “PD-L1 Inhibitors[Title/Abstract]” OR “PD L1 Inhibitors[Title/Abstract]” OR “PD-L1 Inhibitor[Title/Abstract]” OR “PD L1 Inhibitor[Title/Abstract]” OR “Programmed Death-Ligand 1 Inhibitors[Title/Abstract]” OR “Programmed Death Ligand 1 Inhibitors[Title/Abstract]” OR “PD-1-PD-L1 Blockade[Title/Abstract]” OR “Blockade, PD-1-PD-L1[Title/Abstract]” OR “PD 1 PD L1 Blockade[Title/Abstract]” OR “PD-1 Inhibitors[Title/Abstract]” OR “PD 1 Inhibitors[Title/Abstract]” OR “PD-1 Inhibitor[Title/Abstract]” OR “Inhibitor, PD-1[Title/Abstract]” OR “PD 1 Inhibitor[Title/Abstract]” OR “Programmed Cell Death Protein 1 Inhibitor[Title/Abstract]” OR “Programmed Cell Death Protein 1 Inhibitors[Title/Abstract]” |
| **2** | “nivolumab”[Title/Abstract] OR “pembrolizumab”[Title/Abstract] OR “atezolizumab”[Title/Abstract] OR “durvalumab”[Title/Abstract] OR “avelumab”[Title/Abstract] OR “ipilimumab”[Title/Abstract] OR “cemiplimab”[Title/Abstract] OR “Tislelizumab”[Title/Abstract] OR “camrelizumab”[Title/Abstract] OR “toripalimab”[Title/Abstract] |
| **3** | 1 or 2 |
| Part II: Cancer | |
| **4** | “Carcinoma, Non-Small-Cell Lung”[Mesh] OR “Lung Carcinoma, Non-Small-Cell”[Title/Abstract] OR “Non-Small-Cell Lung Carcinomas”[Title/Abstract] OR “Non-Small Cell Lung Cancer”[Title/Abstract] |
| Part III: immune-related adverse events | |
| **5** | "immune-related"[Title/Abstract] AND ("adverse"[Title/Abstract] OR "adversely"[Title/Abstract] OR "adverses"[Title/Abstract]) AND ("event"[Title/Abstract] OR "events"[Title/Abstract] |
| Part IV: Part I, II, AND III | |
| **6** | 3 and 4 and 5 |

| **Web of Science** | #1: TS=(immune checkpoint inhibitor OR immune checkpoint inhibitors OR ICI OR immune checkpoint blockade OR ICB)  #2: TS=( Non-Small-Cell Lung Carcinomas OR Non-Small Cell Lung Cancer)  #3: TS=(immune-related adverse event)  #4: #1 AND #2 AND #3 |
| --- | --- |
| **The Cochrane Library** | #1: MeSH descriptor: [Immune checkpoint inhibitor] explode all trees  #2: MeSH descriptor: [Carcinoma, Non-Small-Cell Lung] explode all trees  #3: MeSH descriptor: [immune related adverse events] explode all trees  #4: (Programmed Death Ligand 1 Inhibitors OR Programmed Death-Ligand 1 Inhibitors OR PD-L1 Inhibitors OR PD L1 Inhibitors OR PD L1 Inhibitor OR PD-L1 Inhibitor OR Checkpoint Inhibitors OR Immune Checkpoint Inhibitor OR Immune Checkpoint Blockers OR Checkpoint Inhibitor, Immune OR Checkpoint Blockers, Immune OR Immune Checkpoint Blockade OR Immune Checkpoint Inhibition OR Checkpoint Blockade, Immune OR Checkpoint Inhibition, Immune OR PD-1 Inhibitors OR PD-1 Inhibitor OR PD 1 Inhibitor OR Programmed Cell Death Protein 1 Inhibitor OR Inhibitor, PD-1 OR Programmed Cell Death Protein 1 Inhibitors OR PD 1 Inhibitors OR PD-1-PD-L1 Blockade OR Blockade, PD-1-PD-L1 OR PD 1 PD L1 Blockade):ti,ab,kw (Word variations have been searched)  #5: (Non-Small-Cell Lung Carcinomas OR Lung Carcinomas, Non-Small-Cell OR Nonsmall Cell Lung Cancer OR Non-Small Cell Lung Carcinoma OR Carcinomas, Non-Small-Cell Lung OR Carcinoma, Non Small Cell Lung OR Lung Carcinoma, Non-Small-Cell OR Non Small Cell Lung Carcinoma OR Non-Small-Cell Lung Carcinoma OR Non-Small Cell Lung Cancer):ti,ab,kw (Word variations have been searched)  #6: #1 OR #3  #7: #2 OR #4  #8: #3 AND #6 AND #7 |
