# Supplementary material for: Immune-related adverse events and their effects on survival outcomes in patients with non-small cell lung cancer treated with immune checkpoint inhibitors: a systematic review and meta-analysis
Source: Front Oncol. 2024 Jun 3;14:1281645. doi: 10.3389/fonc.2024.1281645 (PMC11180722; doi:10.3389/fonc.2024.1281645)
Supplement: Supplementary file 2 [file Table_2.doc]

**Table S2 Summary of different irAEs and efficacy of all included cohort studies**

| Author | irAEs type | | | | | Overall survival  hazard  ratio (95% CI) | | | | | Progression-free survival hazard  ratio (95% CI) | | | | |
| --- | --- | --- | --- | --- | --- | --- | --- | --- | --- | --- | --- | --- | --- | --- | --- |
|  | Pulmonary(%) | Gastrointestinal (%) | Endocrine(%) | Skin(%) | Hepatobiliary(%) | Pulmonary | Gastrointestinal | Endocrine | Skin | Hepatobiliary | Pulmonary | Gastrointestinal | Endocrine | Skin | Hepatobiliary |
| Koji Haratani  2017 | 4.0 | 9.0 | 8.0 | 32.0 | 5.0 | NA | NA | 0.50 (0.03-2.63) | 0.21 (0.05-0.62) | NA | NA | NA | 0.23 (0.03-0.84) | 0.476(0.23-0.91) | NA |
| J.C. Osorio  2017 | NA | NA | 21 | NA | NA | NA | NA | 0.29(  0.09-0.94)- | NA | NA | NA | NA | 0.58(0.27-1.21) | NA | NA |
| Doran Ksienski 2018 | 7.7 | 8.8 | 15.8 | 15.1 | 1.1 | NA | NA | NA | NA | NA | NA | NA | NA | NA | NA |
| R.Dupont 2019 | 2.6 | 9.9 | 6.3 | 18.9 | 1.6 | NA | 0.70 (0.53-0.93) | 0.58 (0.41-0.84) | 0.28 (0.16-0.50) | NA | NA | NA | 0.36(0.26-0.50) | 0.24 (0.16-0.37) | NA |
| Dwight H. Owen 2018 | 33.3 | 11.1 | 25.9 | 22.2 | 7.4 | NA | NA | NA | NA | NA | NA | NA | NA | NA | NA |
| Yukihiro TOI  2018 | 18.0 | 7.0 | 25.0 | 79.0 | 4.0 | NA | NA | NA | NA | NA | NA | NA | NA | NA | NA |
| Wenxian Wang 2021 | 9.5 | 0.9 | 5.4 | 8.1 | 7.7 | NA | NA | NA | NA | NA | NA | NA | NA | NA | NA |
| Koichi Sato 2017 | 45.4 | NA | 34.8 | 9.9 | 9.9 | NA | NA | NA | NA | NA | 0.28 (0.04-1.46) | NA | NA | NA | NA |
| Lea Daniello 2021 | 4.4 | 4.1 | 4.9 | 2.6 | 3.7 | 0.78(0.54-1.14) | 0.78(0.66-0.94) | 0.65(0.48-0.88) | 0.38( 0.27-0.56) | 0.99(0.75-1.29) | NA | NA | NA | NA | NA |
| Biagio Ricciuti 2018 | NA | 28.5 | 30.2 | 21.9 | 33.8 | 0.46 (0.24-0.89) | 0.5 (0.26-0.98) | 0.45 (0.28-0.72) | 0.8 (0.46-1.39) | 0.94 (0.53-1.66) | 0.56 (0.33-0.96) | 0.52 (0.30-0.90) | 0.59 (0.40-0.89) | 0.57 (0.35-0.95) | 0.72 (0.41-1.24) |
| Ana Ortega-Franco 2022 | NA | NA | NA | NA | NA | NA | NA | NA | NA | NA | NA | NA | NA | NA | NA |
| David Conde-Estévez 2021 | 2.9 | 7.1 | 7.1 | 21.4 | 4.3 | 0.83 (0.56 -1.23) | 0.82 (0.48 -1.39) | 0.63 (0.40 -0.98) | 0.44 (0.18 -1.06) | 0.43 (0.15 -1.25) | 0.74 (0.41 -1.31) | 0.79 (0.55 -1.13) | 0.58 (0.23 -1.49) | 0.68 (0.41 -1.14) | 0.85 (0.50 -1.42) |
| Denis Maillet 2020 | NA | NA | NA | NA | NA | NA | NA | NA | NA | NA | NA | NA | NA | NA | NA |
| Yahua Wu 2022 | 22.8 | 4.0 | 12.9 | 5.9 | 8.9 | NA | NA | NA | NA | NA | NA | NA | NA | NA | NA |
| Nadia Guezour2022 | 31 | 22 | 3 | 8 | 11 | NA | NA | NA | NA | NA | NA | NA | NA | NA | NA |
| Fernando C. Santini 2017 | 16.0 | 18.0 | 5.0 | 13.0 | 5.0 | NA | NA | NA | NA | NA | NA | NA | NA | NA | NA |
| J. Rogado 2018 | 3.7 | 0.9 | 21.4 | 2.7 | 1.8 | NA | NA | NA | NA | NA | NA | NA | NA | NA | NA |
| Kim 2017 | NA | NA | 32.7 | NA | NA | NA | NA | 0.11 (0.01–0.92) | NA | NA | NA | NA | 0.38 (0.17–0.85) | NA | NA |
| Ahn 2019 | NA | NA | 5.1 | 9.4 | NA | 4.18(1.42–11.9) | NA | 0.26(0.05–1.29) | 0.42 (0.16–1.09) | NA | NA | 1.69 (0.62–4.60) | 0.37(0.13–1.03) | 0.64(0.35–1.18) | NA |
| Bjørnhart 2019 | NA | NA | NA | NA | NA | NA | NA | NA | NA | NA | NA | NA | NA | NA | NA |
| Cortellini, 2019 | 4.0 | 9.0 | 13 | 10 | 2.0 | 1.32 (0.79–2.19) | 0.61 (0.38–0.98) | 0.55 (0.37–0.83) | 0.43 (0.27–0.70) | 1.09 (0.48–2.45) | 1.20 (0.76–1.92) | 0.68 (0.47–1.01) | 0.63 (0.45–0.89) | 0.46 (0.31–0.69) | 1.47 (0.72–2.96) |
| Grangeon 2018 | 2.0 | 4.0 | 20 | NA | 3.0 | 1.42 (0.45–1.54) | 0.24 (0.03–1.73) | 0.46 (0.25–0.86) | NA | 0.97 (0.30–3.08) | 1.19 (0.52–2.7) | 0.73 (0.35–1.50) | 0.58 (0.39–0.85) | NA | 0.94 (0.45–2.08) |
| Lee 2023 | NA | NA | NA | NA | NA | NA | NA | NA | NA | NA | NA | NA | NA | NA | NA |
| Lesueur 2018 | NA | NA | NA | NA | NA | NA | NA | NA | NA | NA | NA | NA | NA | NA | NA |
| Lisberg2018 | NA | NA | NA | NA | NA | NA | NA | NA | NA | NA | NA | NA | NA | NA | NA |

NA, not applicable
